# Evaluation of left pulmonary artery sling, associated cardiovascular anomalies, and surgical outcomes using cardiovascular computed tomography angiography

**DOI:** 10.1038/srep40042

**Published:** 2017-01-05

**Authors:** Jiajun Xie, Yu-Hsiang Juan, Qiushi Wang, Jimei Chen, Jian Zhuang, Zhaofeng Xie, Changhong Liang, Yulei Zhu, Zhuliang Yu, Jinglei Li, Sachin S. Saboo, Hui Liu

**Affiliations:** 1 Department of Radiology, Guangdong General Hospital, Guangdong Academy of Medical Sciences, Guangzhou, Guangdong, China; Guangdong Cardiovascular Institute, Guangdong Provincial Key Laboratory of South China Structural Heart Disease, Guangdong General Hospital, Guangdong Academy of Medical Sciences, Guangzhou, Guangdong, China; 2 Department of Medical Imaging and Intervention, Chang Gung Memorial Hospital, Linkou and Chang Gung University, Taoyuan, Taiwan; 3 Department of Cardiovascular Surgery, Guangdong Cardiovascular Institute, Guangdong Provincial Key Laboratory of South China Structural Heart Disease; Guangdong General Hospital, Guangdong Academy of Medical Sciences, Guangzhou, Guangdong, China; 4 Department of Pediatric Cardiology, Guangdong Cardiovascular Institute, Guangdong Provincial Key Laboratory of South China Structural Heart Disease; Guangdong General Hospital, Guangdong Academy of Medical Sciences, Guangzhou, Guangdong, China; 5 School of Automation Science and Engineering, South China University of Technology, Guangzhou, Guangdong, China; 6 Department of Radiology, University of Texas Southwestern Medical Center, Dallas, TX, USA

## Abstract

We evaluated the prevalence, image appearance, associated cardiovascular anomalies, and surgical outcomes of left pulmonary artery sling (LPAS) using cardiovascular computed tomography angiography (CCTA). A retrospective search of patients from our database between October 2007 and December 2014 identified 52,200 patients with congenital heart diseases (CHD) referred for CCTA, echocardiography, or magnetic resonance imaging. Clinical information, CCTA findings, associated cardiovascular anomalies, and surgical outcomes were analyzed. We showed a hospital-based prevalence of 71 patients with LPAS (0.14%, 71/52,200) among CHD patients. Of these, 47 patients with CCTA examinations were assessed further. Most patients (40/47, 85%) had associated cardiovascular anomalies, of which ventricular septal defects (22/47, 47%), atrial septal defects (20/47, 43%), patent ductus arteriosus (16/47, 34%), persistent left superior vena cava (14/47, 30%), and abnormal branching of the right pulmonary artery (ABRPA) (14/47, 30%) were most commonly identified. In total, 28 patients underwent LPA reanastomosis and/or tracheoplasty in our center, and 5 died. LPAS had a hospital-based prevalence of 0.14% among CHD patients. ABRPA is not uncommon and must be recognized. CCTA is a feasible method for demonstrating LPAS and its associated cardiovascular anomalies for an optimal pre-operative assessment of LPAS.

Since first described by Glaevecke and Doehle in 1897^1^, left pulmonary artery sling (LPAS) remains a rare congenital cardiovascular anomaly, characterized by the left pulmonary artery (LPA) arising from the right pulmonary artery (RPA) and coursing between the trachea and esophagus to the left pulmonary hilum. Patients with LPAS may experience early onset shortness of breath or dysphagia secondary to the compression of the tracheobronchial tree and esophagus by the aberrant LPA. LPAS is frequently associated with tracheobronchial, intracardiac and other extracardiac abnormalities^2^,^3^.

Cardiovascular computed tomography angiography (CCTA) is accepted as one of the imaging techniques for the evaluation of congenital heart disease (CHD) and is particularly beneficial for depicting both cardiovascular and tracheobronchial anomalies for surgical planning^4^,^5^,^6^.

Previous studies have reported that tracheobronchial anomalies have a greater impact on the patients’ prognosis compared with cardiac defects^7^,^8^. Minor cardiovascular anomalies, such as abnormal branching of the right pulmonary artery (ABRPA), can go unnoticed by both radiologists and cardiologists, and their clinical importance was also been inadequately emphasized in prior reports on LPAS. However, recognition of such imaging findings is critical for safe surgical separation of the anomalous pulmonary arteries when treating patients with LPAS. Thus, we conducted this study based on the CCTA manifestations of LPAS to analyze the prevalence of associated cardiovascular anomalies differences between the types of LPAS, and surgical outcomes.

## Materials and Methods

### Patient selection and clinical information

In this institutional review board approved study (Research Ethics Committee, Guangdong General Hospital, Guangdong Academy of Medical Sciences: No. GDREC2014283H), we conducted a retrospective search for patients with LPAS from our departmental database, which consists of all patients referred for CCTA, echocardiography, or magnetic resonance imaging (MRI) due to CHD. Patients with CCTA examinations were recruited for further studies. All methods were performed in accordance with the relevant guidelines and regulations, and the institutional review board waived the need for informed consent. We recorded and summarized clinical information, including sex, age, presenting symptoms, preoperative ventilation duration, imaging modalities, surgical treatment and outcomes of the patients with LPAS.

### Imaging protocols and post-processing methods

Because of the long study period of 7 years, the patients had undergone two different CCTA scanning protocols using two different CT scanners, as detailed in a previous report^9^.

### Clinical information, image analysis and estimated radiation dosage

The prevalence of LPAS was calculated as the number of patients with LPAS among all patients with CHD evaluated by CCTA, echocardiography or MRI during the same period at our tertiary center.

Transthoracic echocardiography (TTE) images were reviewed by a cardiologist, while the CCTA images were interpreted by two radiologists in consensus from our group. The classification of LPAS was according to the method proposed by Wells *et al*.^2^. According to the differences in tracheobronchial anomalies, LPAS is divided into two main types based on the thoracic level of the carina (type 1: T4-5, type 2: T6-7) and into two subtypes based on the presence or absence of a right upper lobe bronchus. The associated congenital cardiovascular anomalies were analyzed using the CCTA images.

The estimated effective dosages in millisieverts (mSv) for these patients were calculated based on the patients’ age^10^.

### Statistical analysis

Descriptive statistics were used to describe the frequencies (percentage) of each anomaly. The χ^2^ test or Fisher’s exact test was used to compare the presence or absence of the associated cardiovascular anomalies between the two types of LPAS, where appropriate. A *p*-value of <0.05 was considered to indicate statistical significance. Statistical analyses were performed using the SPSS software (ver. 17; SPSS Inc., Chicago, IL, USA).

## Results

### Demographic data and hospital-based prevalence of LPAS

In total 52,200 patients with CHD underwent CCTA, MRI, or echocardiography examinations between October 2007 and December 2014, and 71 patients were diagnosed as having LPAS according to typical CCTA or echocardiography appearances (no MRI was performed for the LPAS cases). Among the 52,200 patients with CHD, 33,363 received surgery or other interventions in our 2,900-bed tertiary referral general hospital. During this period, 11.6% of the patients (6075/52,200) received CCTA examinations for further evaluation. Thus, a hospital-based prevalence of 0.14% (71/52,200) was found among all patients with CHD in our cohort. Among the 71 cases of LPAS, 24 who were diagnosed by echocardiography alone did not receive surgery or another intervention at our hospital and were lost to follow-up. Thus, we only included the patients who underwent CCTA (47 cases) in the analyses. Demographic data and clinical information for these 47 patients are summarized in Table 1. The median age of the patients was 5 months old (range, 1 day to 60 months).

TTE was performed in 40 of the 47 patients with LPAS, while 13 of these patients were diagnosed improperly. Among these 13 cases, 10 cases were inadequately seen (10/40, 25%). Other than LPAS, these 10 patients also had other coexisting findings, comprising of ASD and PLSVC in one patient; PDA in the second patient; VSD, ASD and PAH in the third patient; PFO in the fourth patient; ASD in the fifth patient; DORV, ASD, VSD and PS in the sixth patient; TGA, RAA and PS in the seventh patient; PDA and ASD in the eighth patient; TOF in the ninth patient; VSD and ASD in the tenth patient. Two cases were misdiagnosed as normal (2/40, 5%), and one case misdiagnosed with LPA hypoplasia (1/40, 3%)

### Types of tracheobronchial abnormalities based on CCTA and estimated radiation exposure

**Figure 1:**
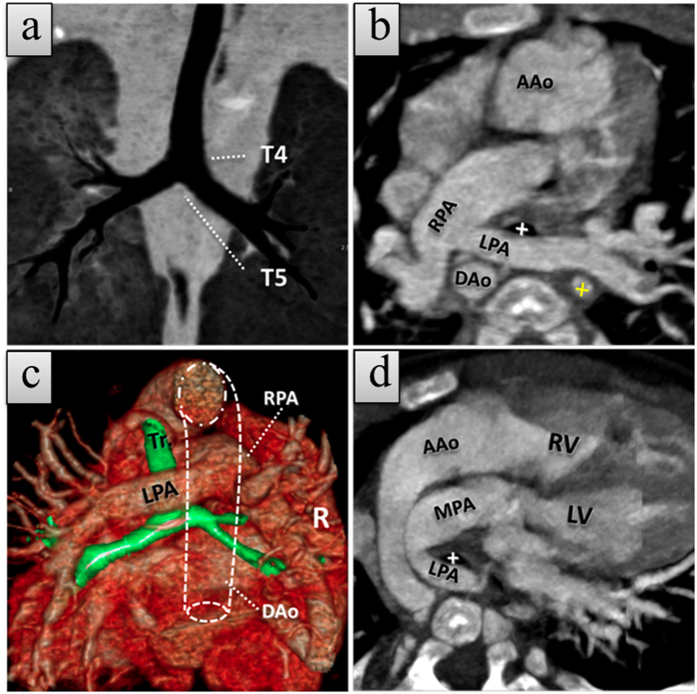
A 1-month-old girl with type 1A left pulmonary artery sling (LPAS) with dextro-transposition of the great arteries (d-TGA). (**a**) Coronal minimum-intensity projection (MinIP) CCTA image shows a normal tracheobronchial branch pattern. (**b, c**) Axial thin slice maximum-intensity projection (MIP) and posterior view of merged volume render CCTA images show anomalous left pulmonary artery (LPA) rising from the proximal right pulmonary artery (RPA) and coursing between the trachea (white cross in **b** and Tr in **c**) and esophagus (cross in yellow). The esophagus lumen showed a high density due to presence of a nasogastric tube. (**d**) Oblique thin slice MIP CCTA image demonstrates ascending aorta (AAo) rising from the right ventricle (RV) and the main pulmonary artery (MPA) from the left ventricle (LV), indicating the d-TGA. Luminal dot line = descending aorta. The patient also has an atrial septal defect and ventricular septal defect (not shown).

**Figure 2:**
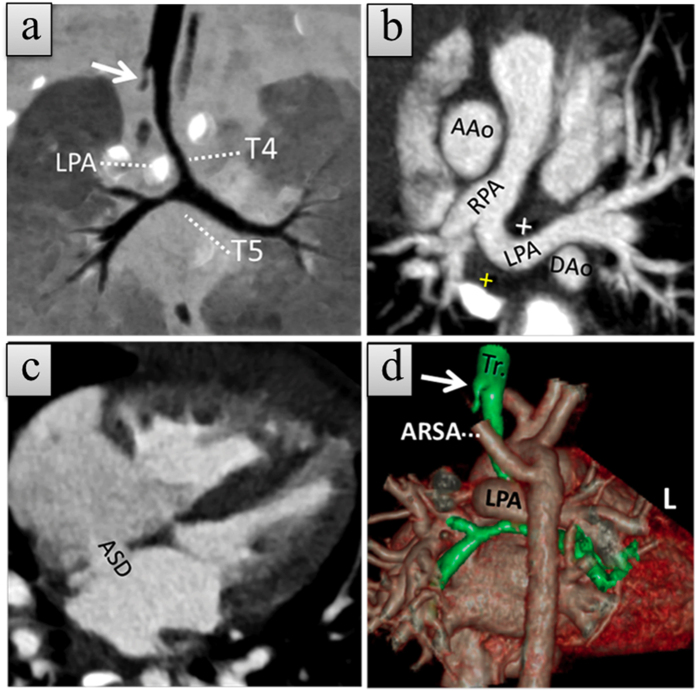
A 9-month-old girl with type 1B left pulmonary artery sling (LPAS) with atrial septal defect (ASD) and aberrant right subclavian artery (ARSA). (**a**) Coronal minimum-intensity projection (MinIP) CCTA image shows a normal level of bronchial bifurcation but with a right rudimentary tracheal bronchus (white arrow) and an abnormal course of the left pulmonary artery (LPA). (**b,c**), Axial thin slice maximum-intensity projection (MIP) CCTA images demonstrate ASD and anomalous LPA originating from the proximal right pulmonary artery (RPA) and coursing between the trachea (cross in white) and esophagus (cross in yellow). **(d**) Posterior view of the trachea (Tr), and cardiovascular merged volume render CCTA image reveals rudimentary right rudimentary tracheal bronchus (white arrow) and ARSA arising from the aortic isthmus. The patient also has a ventricle septal defect (not shown).

**Figure 3:**
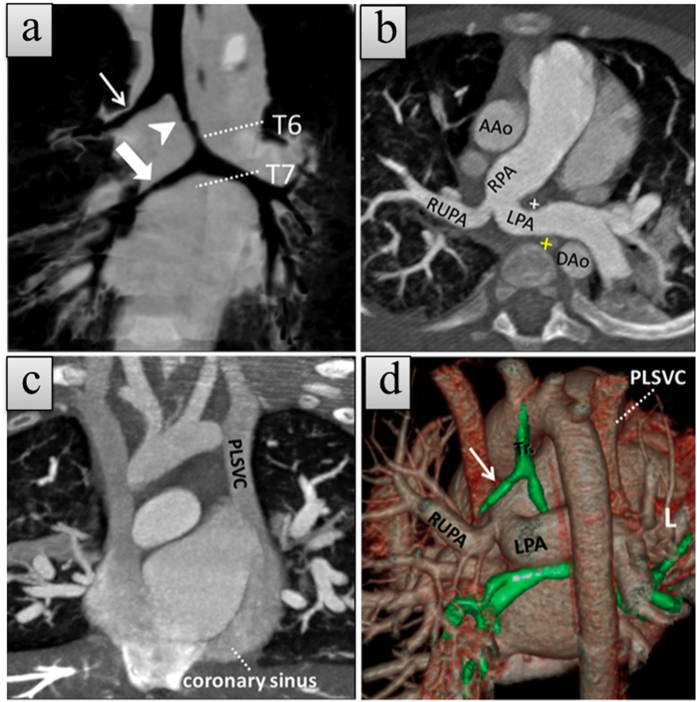
A 6-month-old boy with type 2A left pulmonary artery sling (LPAS) with persistent left superior vena cava (PLSVC). (**a**) Coronal minimum-intensity projection (MinIP) CCTA image shows a lower level of bronchial bifurcation with a classic tracheal bronchus (white arrow), left intermediate bronchus (arrowhead), and bridging bronchus (broad arrow). (**b**) Axial thin slice maximum-intensity projection (MIP) CCTA image shows anomalous left pulmonary artery (LPA) arising from the proximal right pulmonary artery (RPA) and coursing between the trachea (cross in white) and esophagus (cross in yellow). The esophagus lumen showed a high density due to a nasogastric tube. Right upper pulmonary artery (RUPA) can be seen originating from the common orifice of LPA and RPA; (**c**) Coronal thin slice MIP CCTA image demonstrates PLSVC draining into the right atrium via the coronary sinus. (**d**) Posterior view of trachea (Tr) and cardiovascular merged volume render CCTA image shows tracheal bronchus (white arrow) and anomalous LPA course. The patient also has atrial septal defect and ventricular septal defects (not shown).

**Figure 4:**
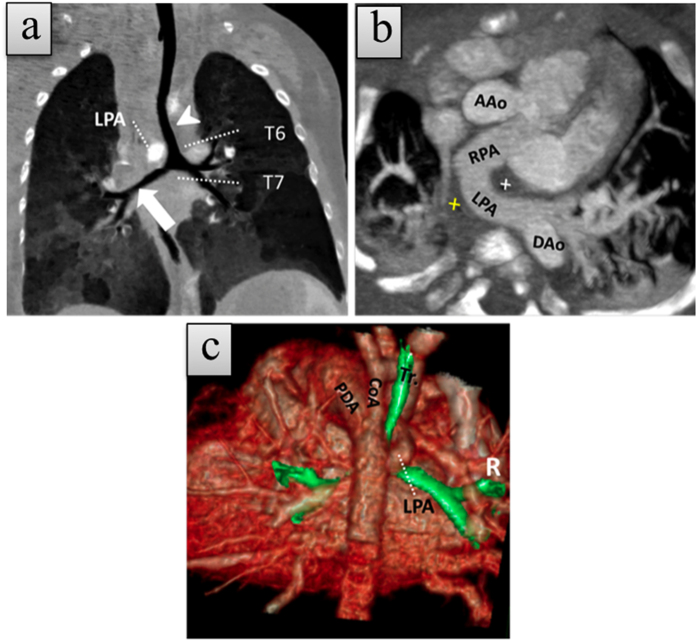
1-month-old girl with type 2B left pulmonary artery sling (LPAS) with coarctation of aorta (CoA) and patent ductus arteriosus (PDA). (**a**) Coronal thin slice minimum-intensity projection (MinIP) CCTA image shows a lower level of bronchial bifurcation with classic left intermediate bronchus (arrowhead) and bridging bronchus (broad arrow). (**b**) Axial thin slice maximum-intensity projection (MIP) CCTA image reveals anomalous LPA originating from the proximal RPA and coursing between the trachea (cross in white) and esophagus (cross in yellow). (**c**) Volume render CCTA image shows an abnormal course of the LPA, a big PDA, and CoA.

According to method of Wells classification for LPAS [2], there were 20 patients (20/47, 43%) with type 1 (type 1A: 14 patients and type 1B: 6 patients, Figs 1 and 2) and 27 patients (27/47, 57%) with type 2 manifestations (type 2A: 14 patients and type 2B: 13 patients, Figs 3 and 4) in our series.

The median and range values of the estimated effective radiation dosages using the 64- and 256-slice CT scanners were 5.09 mSv (3.42 mSv–7.67 mSv) and 1.14 mSv (0.42 mSv–2.77 mSv) in their respectively age groups.

### Associated congenital cardiovascular anomalies based on CCTA

Of the patients with LPAS in our series, 85% (40/47) had other associated congenital cardiovascular anomalies. The distribution of associated CHD is listed in Table 2. Ventricular septal defect (VSD; 22/47, 47%) was the most commonly found associated anomaly in LPAS patients, followed by atrial septal defect (ASD; 20/47, 43%), patent ductus arteriosus (PDA; 16/47, 34%, including 5 neonates), persistent left superior vena cava (PLSVC; 14/47, 30%), ABRPA (14/47, 30%), coarctation of the aorta (CoA; 6/47, 13%), and abnormalities of aortic arch branching (6/47, 13%). Most of the associated anomalies were seen more with type 2 than type 1 LPAS, but there was no significant difference, with the exception of ABRPA (2 cases with type 1 vs. 12 cases with type 2, *p* = 0.011).

Abnormalities of aortic arch branching, including aberrant right subclavian artery (3 cases, 6%), aberrant left subclavian artery (1 case), common trunk with left innominate artery and left common carotid artery (1 case), and left vertebral artery originating from the aortic arch (1 case), seemed to be more common in patients with type 2 than type 1 LPAS (Table 2). Another five associated CHD were also found: one involved the anomalous origin of the right coronary artery and partial anomalous pulmonary venous connection (PAPVC), one involved the transposition of great arteries (TGA) associated with the right arch and right descending aorta (Fig. 1), one involved the double-outlet right ventricle (DORV) associated with pulmonary stenosis, and one involved the tetralogy of Fallot (TOF).

### Abnormal branching of the right pulmonary artery

**Figure 5:**
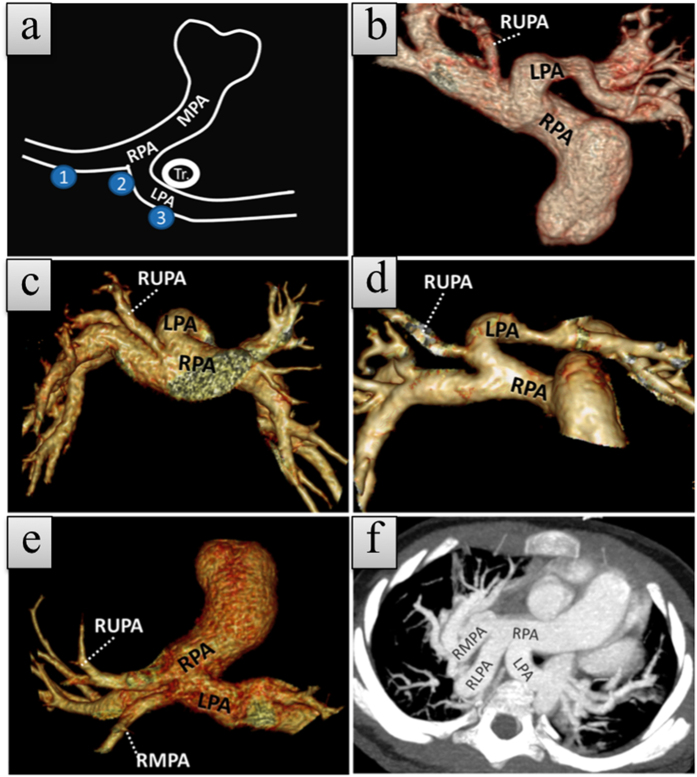
Volume rendering (B–E) and maximum-intensity-projection (MIP, F) images show normal and anomalous origin of the proximal branches of the right pulmonary artery (RPA) in five patients with left pulmonary artery sling. (**a**) Schematic diagram shows the origin sites of proximal right pulmonary artery branches: RPA, common orifice, left pulmonary artery (LPA). (**b–e**) Volume-rendered images show the origins of the proximal branches of the RPA in our series. (**b**) RUPA originates from the RPA, (**c**) RUPA originates from the common orifice of the LPA and RPA, (**d**) RUPA originates from the LPA, (**e**) RMPA originates from the LPA and inferior to the RUPA origin site, and (**f**) MIP image shows RLPA originating from the common orifice of the LPA and RPA.

We reviewed all of the proximal branches of the RPA on CCTA images, and the results are summarized in Table 3. The results showed that the right upper pulmonary artery (RUPA) could originate from the RPA, the common orifice just between the LPA and RPA and even the LPA (35/47, 74%, 2/47, 4%, and 10/47, 21% respectively, Fig. 5B–D). There were more patients with type 2 than type 1 LPAS (8 and 2 patients, respectively) with an anomalous origin of the RUPA from LPA, but the difference was not statistically significant, probably due to the small sample numbers.

In two other ABPRA cases, one showed an anomalous origin of the right middle pulmonary artery (RMPA) (Fig. 5e) from the LPA, and the other showed an anomalous origin of the right lower pulmonary artery (RLPA) from the common orifice of the LPA and RPA (Fig. 5f).

### Surgical treatment, clinical outcomes and follow-up

In total 28 (28/47, 60%) patients underwent surgery to correct the cardiovascular anomalies or tracheal abnormalities in our series, including 13 type 1 and 15 type 2 patients (46% vs 54%). LPA reimplantation was performed in 27 patients (27/28), including two patients with accompanying tracheoplasties (one patch augmentation and one sliding tracheoplasty), while one patient had only a sliding tracheoplasty. These three patients receiving tracheal surgeries were all type 2 cases. Other associated procedures (such as correct or palliative operations) were also performed for these patients to correct other associated cardiovascular anomalies, including ASD closure, VSD repair, PDA ligation, right ventricular outflow tract patch expansion. pulmonary artery (PA) banding, and a bidirectional Glenn shunt. Among the five deaths, three were early deaths due to severe hypoxic ischemic encephalopathy (1 patient) and aggravation of preexisting respiratory failure (2 patients), and the other two deaths occurred later due to airway obstruction by repetitive granulation tissue growing after the tracheoplasty.

In the 23 living patients, including one patient who underwent tracheal surgery, their respiratory symptoms improved over an average of 20 days of postoperative hospitalization, and the patients were well at 1-year follow-up visit.

## Discussion

Yu *et al*.^11^ evaluated the prevalence of LPAS in a large-scale screening study using 2D echocardiography, and found a prevalence of 59 per million school-aged children. Our cohort complemented the study by Yu *et al*., showing a hospital-based prevalence of 0.14% (71/52,200) among patients with CHD.

Gikonyo *et al*.^3^ reviewed the clinical symptoms of LPAS and reported that LPAS usually causes airway obstruction before 1 year of age in ~90% of cases; however, 14 (12%) of the 130 patients surveyed were asymptomatic. Our cohort showed similar results in that 10% (5/47) of patients were asymptomatic, while 89% (42/47) had associated clinical manifestations, with an median onset age of 5 months (79% presenting with shortness of breath, and 36% required preoperation ventilation; dysphagia occurred in 23%, Table 1).

According to the classification method for the tracheobronchial tree proposed by Wells *et al*.^2^, tracheal bifurcation of type 1 LPAS is located at the T4-5 level just above the carina, while type 2 is at the T6-7 level. Type 2 LPAS is associated with left intermediate bronchus and bridging bronchus and the location at T6-7 is more commonly associated with longer tracheobronchial stenosis which increases respiratory difficulties.

The proportion of type 1 LPAS in our cohort (43%, 20/47) is similar to that reported by Zhong *et al*. (48%, 13/27)^12^ using the same classification scheme. Type 1A refers to a normal tracheobronchial branch pattern and was seen in 30% (14/47) of patients in our cohort, also similar to the results of Zhong *et al*. (8/27, 30%). Other types such as type 1B, 2A and 2B are abnormal tracheobronchial branch patterns. Among these, type 1B is similar to type 1A but has a true right tracheal bronchus or tracheal diverticulum. Type 2 LPAS was more common; our results were consistent with the results of Wells *et al*. and Lee *et al*.^2^,^13^. We also observed more operative mortalities among type 2 versus type 1 due to poor preoperative airway conditions (4 of 5 deaths were type 2 LPAS in our series).

Our study showed that VSD (22/47, 47%), ASD (20/47, 43%), PDA (16/47, 34%, including 5 neonates, all without prostaglandin therapy) and PLSVC (14/47, 30%) were common findings in LPAS patients. These results are much higher than those reported in a case review by Gikonyo *et al*., which showed remarkably lower proportions of VSD (12/93, 13%), ASD (9/93, 10%), PDA (4/93, 4%), and PLSVC (8/93, 9%)^3^. However, our results were more similar to the CCTA study of Chen *et al*. from Taiwan: VSD (4/18, 22%), ASD (6/18, 33%), PDA (7/18, 39%), and PLSVC (4/18, 22%)^14^.

Our results showed that ABRPA is not an uncommon condition in patients with LPAS (14/47, 30%). However, to our knowledge, a RUPA anomaly originating from the LPA has only been reported in one case report^15^ from 1977, and no case of a right middle or lower pulmonary artery (RMPA) anomaly originating from LPA has been reported in the English language literature. In addition to the lack of emphasis in prior reports, ABRPA may be overlooked by both radiologists and cardiac surgeons. When ABRPA is unrecognized or misdiagnosed as a part of the RPA during surgery, cardiac surgeons may accidentally injure the anomalous origin of the ABRPA, increasing the risk of complications (such as major intraoperative blood loss) or the duration of hospitalization. Thus, pre-operative confirmation of the presence of ABRPA is important when considering surgical treatment of LPAS. For the patients in whom surgery was not performed, CCTA can be an alternative reference standard for the diagnosis of LPAS.

In the present study, complex cardiovascular anomalies were not common in patients with LPAS. Only a few case reports have focused on LPAS with TOF, DORV, or PAPVC^3^,^16^,^17^,^18^. Consistent with previous reports, our study showed one case each with PAPVC and TOF. To our knowledge, the combined finding of TGA with LPAS has not been reported before. The only case of LPAS with TGA in our series was also associated with complex cardiovascular anomalies, including right aortic arch, ASD, VSD and PDA. This patient subsequently underwent LPA reimplantation, PDA suture and PA banding.

Reimplantation of the left pulmonary artery with or without tracheoplasty and simultaneous surgical correction of cardiac defects were performed safely in most of the LPAS patients, with a survival rate of 82% (23/28), mean hospitalization duration of 20 days, and good 1-year follow-up results.

The rate of postoperative mortality in our study was 17.9% (5/28), which is in consistent with other reports on LPAS patients from the past two decades, in which the rates ranged from 11% to 45%^12^,^14^,^19^,^20^,^21^. Yong *et al*.^21^ showed a descending trend for the postoperative mortality every ten years from 1984 to 2011, with an average of ~14.3%. Yong *et al*. also explained that the mortalities were related to the need for tracheal surgery, while those who did not require tracheal surgery had excellent outcomes.

## Limitation

Our study was limited by its retrospective nature and data collection from a single tertiary medical center. Further large-scale prospective studies may allow us to further clarify the differences among the results from our cohort and other prior studies. In addition, although all patients had typical imaging presentations of LPAS on CCTA, only 60% of patients were confirmed by surgery. The patients receiving only echocardiography were lost to follow-up after the initial diagnosis, which limited the total number of LPAS patients to 47 for further analysis. Future studies involving larger cohorts of patients may further confirm the results of our study. For the patients who did not undergo surgery, the diagnosis of LPAS could not be confirmed by surgical findings, despite typical imaging findings on CCTA. Furthermore, LPAS remains a rare congenital anomaly, and due to the high proportion of patients with tracheobronchial anomalies, further studies focusing on characterizing the CCTA findings of the tracheobronchial entities may be beneficial. Finally, the diagnosis of ABRPA is often overlooked, and follow-up study focusing on the diagnosis of ABRPA and the feasibility of CCTA in the pre-operative diagnosis of ABRPA from interobserver comparison may be beneficial in understanding the advantage of CCTA in assisting the diagnosis of ABRPA.

## Conclusions

LPAS had a hospital-based prevalence of 0.14% among patients with congenital heart diseases. The results in this study showed ABRPA is not uncommon and must be recognized. CCTA is an excellent tool for demonstrating LPAS and its associated cardiovascular anomalies, which is crucial information for an optimal preoperative assessment of LPAS.

## Additional Information

**How to cite this article**: Xie, J. *et al*. Evaluation of left pulmonary artery sling, associated cardiovascular anomalies, and surgical outcomes using cardiovascular computed tomography angiography. *Sci. Rep.* **7**, 40042; doi: 10.1038/srep40042 (2017).

**Publisher's note:** Springer Nature remains neutral with regard to jurisdictional claims in published maps and institutional affiliations.

## Figures and Tables

**Table 1:** Demographic data of the 47 patients with left pulmonary artery sling.

Clinical information	Total
Number of patients	47 (100%)
Sex	
Male	28 (60%)
Female	19 (40%)
Age (median, range)	5.0 mo (1 d-60 mo)
Body weight (median, range)	5 kg (1.9–15 kg)
Symptoms and signs	
Asymptomatic cases	5 (11%)
Symptomatic cases	42 (90%)
Shortness of breath or stridor	37 (79%)
Dysphagia	11 (23%)
Pre-operation ventilation	17 (36%)
Operation	28 (60%)
LPA reanastomosis only	25/28 (89%)
LPA reanastomosis + tracheoplasty	2/28 (7%)
Tracheoplasty only	1/28 (4%)
Post-operation follow-up	
Mortalities	5/28 (18%)
Death time (median, range)	5 d (2–97 d)
Survivors follow-up time	23 mo (3–36 mo)

Note: Data represent numbers (percentage). Abbreviations: mo (months), d (days), kg (kilograms).

**Table 2:** Congenital cardiovascular anomalies associated with the 47 cases of LPAS.

Associated anomalies	Type 1 (n = 20)	Type 2 (n = 27)	*p*-value	Total
Ventricular septal defect	12	10	0.119^a^	22(47%)
Atrial septal defect	7	13	0.367^a^	20(43%)
Patent ductus arteriosus	8	8	0.458^a^	16(34%)
PLSVC	6	8	0.978^a^	14(30%)
ABPRA	2	12	0.011^a^	14(30%)
Coarctation of aorta	1	5	0.352^b^	6(13%)
Abnormalities of aortic arch branching	1	5	0.352^b^	6(13%)
Aberrant right subclavian artery	1	2	1.000^b^	3(6%)
Aberrant left subclavian artery	0	1	1.000^c^	1(2%)
Common trunk with left innominate artery and left common carotid artery	0	1	1.000^c^	1(2%)
Left vertebral artery originating from aortic arch	0	1	1.000^c^	1(2%)
AORCA	0	1	1.000^c^	1(2%)
PAPVC	0	1	1.000^c^	1(2%)
TGA + RAA + PS	1	0	0.426^c^	1(2%)
DORV + PS	1	0	0.426^c^	1(2%)
Tetralogy of Fallot	1	0	0.426^c^	1(2%)

Note: Data from the 47 patients with LPAS may not add up to a total 100% because of rounding. The total may be more than 100%, because more than one associated anomaly may have been present in a patient. (a) χ^2^, (b) χ^2^ correction for continuity, and (c) Fisher’s exact tests were used to compared the presence or absence of the associated cardiovascular anomalies between the two types of LPAS. Abbreviations: PLSVC (persistent left superior vena cava), ABRPA (abnormal branching of the right pulmonary artery), AORCA (anomalous origin of right coronary artery), PAPVC (partial anomalous pulmonary venous connection), TGA (transposition of the great arteries), RAA (right-side aortic arch), PS (pulmonary stenosis), DORV (double outlet right ventricle).

**Table 3:** Origins of proximal RPA branches in 47 patients with left pulmonary artery sling.

Origins of proximal RPA branches	Type 1 (n = 20)	Type 2 (n = 27)	Total
Origin of RUPA			
RPA	18	17	35(74%)
Common orifice	0	2	2(4%)
LPA	2	8	10(21%)
Origin of RMPA			
RPA	20	26	46(98%)
Common orifice	0	0	0
LPA	0	1	1(2%)
Origin of RLPA			
RPA	19	27	46(98%)
Common orifice	1	0	1(2%)
LPA	0	0	0

Abbreviations: RUPA (right upper pulmonary artery); RMPA (right middle pulmonary artery), RLPA (right lower pulmonary artery), LPA (left pulmonary artery), RPA (right pulmonary artery).

## References

[b1] Pawade A., de Leval M. R., Elliott M. J. & Stark J. Pulmonary artery sling. Ann Thorac Surg 54, 967–970 (1992). 1417294 10.1016/0003-4975(92)90661-m

[b2] Wells T. R., Gwinn J. L., Landing B. H. & Stanley P. Reconsideration of the anatomy of sling left pulmonary artery: the association of one form with bridging bronchus and imperforate anus. Anatomic and diagnostic aspects. J Pediatr Surg 23, 892–898 (1988). 3069994 10.1016/s0022-3468(88)80379-8

[b3] Gikonyo B. M., Jue K. L. & Edwards J. E. Pulmonary vascular sling: report of seven cases and review of the literature. Pediatr Cardiol 10, 81–89, doi: 10.1007/BF02309919 (1989). 2657677

[b4] Leonardi B. . Imaging modalities in children with vascular ring and pulmonary artery sling. Pediatr Pulmonol 50, 781–788, doi: 10.1002/ppul.23075 (2015). 24979312

[b5] Hu X. H., Pa M. E., Shen Q. L. & Huang G. Y. Multi-detector computed tomography evaluation of tracheobronchial anomaly in pediatric patients with left pulmonary artery sling. Chin Med J(Engl) 126, 2790–2792 (2013). 23876916

[b6] Lambert V. . Preoperative and postoperative evaluation of airways compression in pediatric patients with 3-dimensional multislice computed tomographic scanning: effect on surgical management. J Thorac Cardiovasc Surg 129, 1111–1118, doi: 10.1016/j.jtcvs.2004.08.030 (2005). 15867788

[b7] Yilmaz M. . Vascular anomalies causing tracheoesophageal compression: a 20-year experience in diagnosis and management. Heart Surg Forum 6, 149–152 (2003). 12821429

[b8] Berdon W. E., Muensterer O. J., Zong Y. M. & Backer C. L. The triad of bridging bronchus malformation associated with left pulmonary artery sling and narrowing of the airway: the legacy of Wells and Landing. Pediatr Radiol 42, 215–219, doi: 10.1007/s00247-011-2273-2 (2012). 22002862

[b9] Liu H. . Anomalous Origin of One Pulmonary Artery Branch From the Aorta: Role of MDCT Angiography. AJR Am J Roentgenol 204, 979–987, doi: 10.2214/AJR.14.12730 (2015). 25905931

[b10] Thomas K. E. & Wang B. Age-specific effective doses for pediatric MSCT examinations at a large children’s hospital using DLP conversion coefficients: a simple estimation method. Pediatr Radiol 38, 645–656, doi: 10.1007/s00247-008-0794-0 (2008). 18392817

[b11] Yu J. M. . The prevalence and clinical impact of pulmonary artery sling on school-aged children: a large-scale screening study. Pediatr Pulmonol 43, 656–661, doi: 10.1002/ppul.20823 (2008). 18484662

[b12] Zhong Y. M. . CT assessment of tracheobronchial anomaly in left pulmonary artery sling. Pediatr Radiol 40, 1755–1762, doi: 10.1007/s00247-010-1682-y (2010). 20490486

[b13] Lee K. H. . Use of imaging for assessing anatomical relationships of tracheobronchial anomalies associated with left pulmonary artery sling. Pediatr Radiol 31, 269–278, doi: 10.1007/s002470000423 (2001). 11321746

[b14] Chen S. J. . Left pulmonary artery sling complex: computed tomography and hypothesis of embryogenesis. Ann Thorac Surg 84, 1645–1650, doi: 10.1016/j.athoracsur.2007.05.094 (2007). 17954077

[b15] Bamman J. L., Ward B. H. & Woodrum D. E. Aberrant left pulmonary artery. Clinical and embryologic factors. Chest 72, 67–71 (1977). 872657 10.1378/chest.72.1.67

[b16] Loukanov T., Sebening C., Springer W., Ulmer H. & Hagl S. Simultaneous management of congenital tracheal stenosis and cardiac anomalies in infants. J Thorac Cardiovasc Surg 130, 1537–1541, doi: 10.1016/j.jtcvs.2005.08.031 (2005). 16307995

[b17] Takeda Y. . Pulmonary artery sling associated with tetralogy of fallot. Asian Cardiovasc Thorac Ann 13, 77–78 (2005). 15793059 10.1177/021849230501300119

[b18] Murdison K. A. & Weinberg P. M. Tetralogy of Fallot with severe pulmonary valvar stenosis and pulmonary vascular sling (anomalous origin of the left pulmonary artery from the right pulmonary artery). Pediatr Cardiol 12, 189–191, doi: 10.1007/bf02238531 (1991). 1876521

[b19] Kwak J. G. . Is tracheoplasty necessary for all patients with pulmonary artery sling and tracheal stenosis? Pediatr Cardiol 34, 498–503, doi: 10.1007/s00246-012-0481-7 (2013). 22890626

[b20] Oshima Y. . Management of pulmonary artery sling associated with tracheal stenosis. Ann Thorac Surg 86, 1334–1338, doi: 10.1016/j.athoracsur.2008.04.020 (2008). 18805188

[b21] Yong M. S. . Surgical management of pulmonary artery sling in children. J Thorac Cardiovasc Surg 145, 1033–1039, doi: 10.1016/j.jtcvs.2012.05.017 (2013). 22698556

